# Comparative Efficacy of Silicone Sheets and Hyperbaric Oxygen Therapy in Post-Surgical Scar Prevention: A Prospective Observational Study

**DOI:** 10.7150/ijms.108397

**Published:** 2025-02-11

**Authors:** Po-Chung Chen, Tzung-Cheng Liao, Chang-Yi Chou, Chi-Ming Chu, Po-Jen Hsiao, Hsieh-Chih Tsai

**Affiliations:** 1 Graduate Institute of Applied Science and Technology, National Taiwan University of Science and Technology, Taipei, Taiwan.; 2 Department of Orthopedics, Taoyuan Armed Forces General Hospital, Taoyuan, Taiwan.; 3 Division of Plastic surgery, Department of Surgery, Taoyuan Armed Forces General Hospital, Taoyuan, Taiwan.; 4 Division of Biostatistics and Medical Informatics, Department of Epidemiology, School of Public Health, National Defense Medical Center, Taipei, Taiwan.; 5 Graduate Institute of Medical Sciences, National Defense Medical Center, Taipei, Taiwan.; 6 Division of Nephrology, Department of Internal Medicine, Taoyuan Armed Forces General Hospital, Taoyuan, Taiwan.; 7 Division of Nephrology, Department of Internal Medicine, Tri-Service General Hospital, National Defense Medical Center, Taipei, Taiwan.; 8 Department of Life Sciences, National Central University, Taoyuan, Taiwan.

**Keywords:** hyperbaric oxygen therapy, silicone sheets, scar prevention, Patient and Observer Scar Assessment Scale (POSAS)

## Abstract

**Background:** Postoperative scarring can significantly impact physical function, aesthetic outcomes, and overall quality of life. Effective scar management is crucial to mitigate these effects. This study aimed to compare the efficacy of silicone sheets (SS) and hyperbaric oxygen therapy (HBOT) in minimizing postoperative scar formation and improving wound healing outcomes.

**Methods:** A total of 40 patients with clean, non-infected linear postoperative wounds were enrolled in a 12-week prospective observational study. Participants were randomly assigned to either the HBOT or SS group. The HBOT group underwent seven sessions of HBOT starting 4 weeks postoperatively, while the SS group applied silicone sheets to the wound from 4 to 12 weeks postoperatively. Scar outcomes were evaluated at 4, 8, and 12 weeks using the Patient and Observer Scar Assessment Scale (POSAS), which measures various parameters, including vascularity, pigmentation, thickness, relief, pliability, surface area and overall opinion.

**Results:** After applying exclusion criteria, 33 patients completed the study, with 18 in the HBOT group and 15 in the SS group. Both interventions significantly improved scar parameters such as vascularity, thickness, relief, pliability, and overall opinion over 12 weeks. SS was particularly effective in reducing pigmentation, while HBOT achieved greater reductions in scar surface area. By the study's conclusion, SS demonstrated superior outcomes in vascularity, pigmentation, scar thickness, relief, pliability, and overall appearance, whereas HBOT excelled in reducing surface area.

**Conclusion:** Both HBOT and SS are effective scar management options, each with unique benefits. Selecting the appropriate treatment based on patient-specific needs and wound characteristics is essential to achieve optimal outcomes and enhance patient satisfaction.

## Introduction

Scarring is an inevitable outcome of surgical procedures and traumatic injuries, often resulting in physical discomfort and adversely impacting patients' quality of life 1, 2. Scars are broadly categorized as immature or mature, with the latter further classified as “normal,” “atrophic,” or “hypertrophic.” Among these, hypertrophic scars and keloids pose significant challenges due to their propensity for recurrence and associated symptoms, such as pain, pruritus, and psychological distress 3. These issues underscore the critical need for effective strategies to mitigate scar formation, given the substantial aesthetic and functional implications. The pathophysiology of scar formation is complex, involving multifactorial processes such as inflammation, collagen deposition, and extracellular matrix (ECM) remodeling 4. The inflammatory phase, characterized by immune cell infiltration and increased vascular permeability, initiates the wound-healing cascade, followed by the proliferative phase, during which fibroblasts synthesize collagen and other ECM components 5. Dysregulation at any stage of this process can lead to pathological scarring, emphasizing the importance of timely and effective therapeutic interventions.

Silicone-based treatments, including silicone gel sheets and gels, are among the most widely adopted methods for scar prevention in clinical practice 6. These products create a protective barrier that maintains optimal hydration at the wound site, facilitating an environment conducive to healing and reducing the risk of aberrant scar formation 7. Silicone therapy typically begins a few weeks postoperatively and is sustained for several months, demonstrating efficacy in preventing hypertrophic scars and keloids 8-11. However, challenges such as patient adherence and discomfort, particularly in highly mobile regions like joints, may limit the practicality of silicone sheet application 8, 12. Hyperbaric oxygen therapy (HBOT) has garnered attention as an alternative modality for enhancing wound healing and minimizing scar development 13-15. HBOT involves administering 100% oxygen in a pressurized chamber, significantly enhancing tissue oxygenation and promoting angiogenesis 16, 17. Beyond oxygenation, HBOT has been shown to modulate inflammatory pathways, improve collagen organization, and accelerate the healing process, potentially reducing scar thickness and enhancing scar quality 18. Nonetheless, its widespread use is constrained by reliance on specialized infrastructure and the need for multiple sessions, posing logistical challenges for patients 19.

Given the respective benefits and limitations of both silicone therapy and HBOT, comparative analyses are essential to evaluate their relative efficacy in scar prevention. This study aimed to systematically compare the effectiveness of silicone sheets (SS) and HBOT in preventing postoperative scar formation. By providing evidence-based insights, this research seeks to guide clinicians in optimizing scar management strategies, aligning treatment approaches with patient needs, wound characteristics, and specific therapeutic goals.

## Methods

### Patients and study design

This 12-week prospective observational study included 40 postoperative patients. Eligibility criteria required participants to have clean, non-infected linear wounds (Class 1 wounds) 20, 21 with a minimum length of 2 cm. Patients with autoimmune diseases, diabetes mellitus, current use of steroids or other medications affecting wound healing, malignant disease, infections, or contraindications to HBOT were excluded 10. Eligible patients were randomly assigned to one of two groups: the HBOT Group or the SS Group.

- **HBOT Group:** Participants underwent seven HBOT sessions starting 4 weeks postoperatively. Each session was conducted once daily, at a pressure of 2.5 atmospheres absolute (ATA), with a duration of 100 min.
- **SS Group:** Participants applied a silicone sheet (FoamLite™; ConvaTec, Singapore) to the wound starting 4 weeks after surgery. The silicone sheet was replaced every 24 h and used continuously until 12 weeks postoperatively.

### Assessment

Clinical outcomes were evaluated using the Patient and Observer Scar Assessment Scale (POSAS) 2.0 Observer Scale (Figure S1) 22. The Observer Scale includes seven parameters: vascularity, pigmentation, thickness, relief, pliability, surface area, and overall opinion. Trained healthcare professionals, including specialized nurses, assessed these parameters at the 4th, 8th, and 12th weeks postoperatively to objectively evaluate scar characteristics.

### Statistical analysis

Descriptive statistics were used to summarize demographic and baseline characteristics. Continuous variables were expressed as mean ± standard deviation 12. Between-group comparisons at the 4th, 8th, and 12th weeks were conducted using independent sample t-tests, while paired sample t-tests assessed within-group changes over time. The *p*-values for each parameter reflect two types of comparisons: within-group comparisons, where the changes in scores at 8 weeks and 12 weeks are compared to the baseline (4 weeks) within each group (HBOT or SS). Between-group comparisons, where the scores at 4 weeks, 8 weeks and 12 weeks are compared between the SS group and the HBOT group at each corresponding time point. Statistical significance was set at *p* < 0.05. Additionally, in order to minimize the impact of baseline differences, percentage improvements in each parameter from 4 weeks to 12 weeks were calculated. The percentage improvement is calculated by determining the change from baseline (4 weeks) to the final assessment (12 weeks) as a percentage of the baseline value. It can provide a clearer picture of the relative treatment effects between the two groups.

## Results

### Participant enrollment

**Figure 1:**
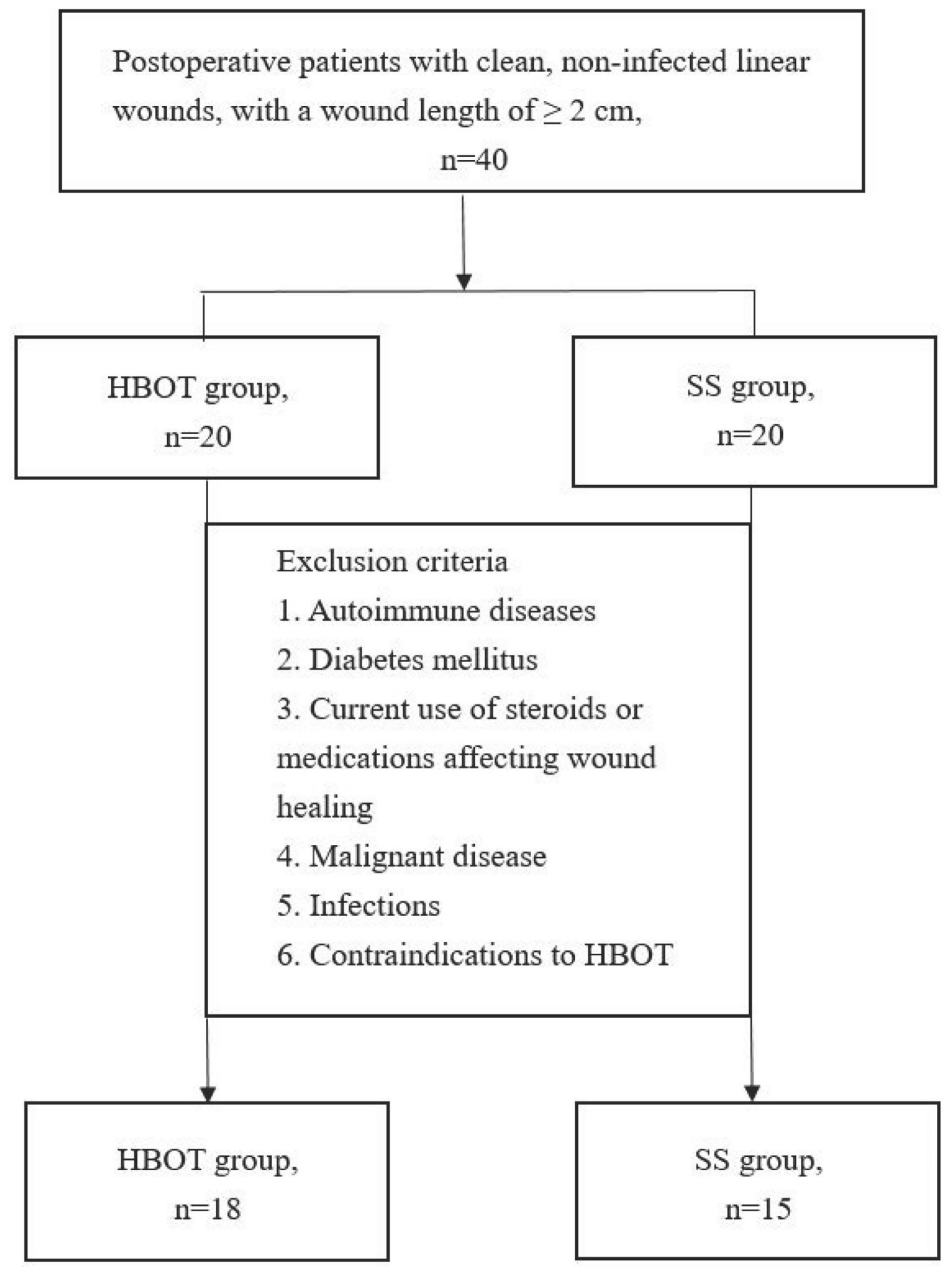
Study enrollment and allocation flowchart for the randomized controlled trial. Of the 40 patients screened, 7 were excluded based on predefined criteria. The remaining 33 participants were randomized into two groups: the hyperbaric oxygen therapy (HBOT) group (n = 18) and the silicone sheets (SS) group (n = 15).

A total of 40 patients were enrolled, 7 patients were excluded due to meeting the exclusion criteria, leaving 18 patients in the HBOT group and 15 patients in the SS group (Figure 1). The mean ages of the HBOT and SS groups were 42.39 and 42.87 years, respectively, with average body mass index of 25.59 kg/m^2^ and 23.94 kg/m^2^. Baseline characteristics were comparable between the two groups. Detailed demographic and baseline data are presented in Table 1.

### POSAS

**Figure 2:**
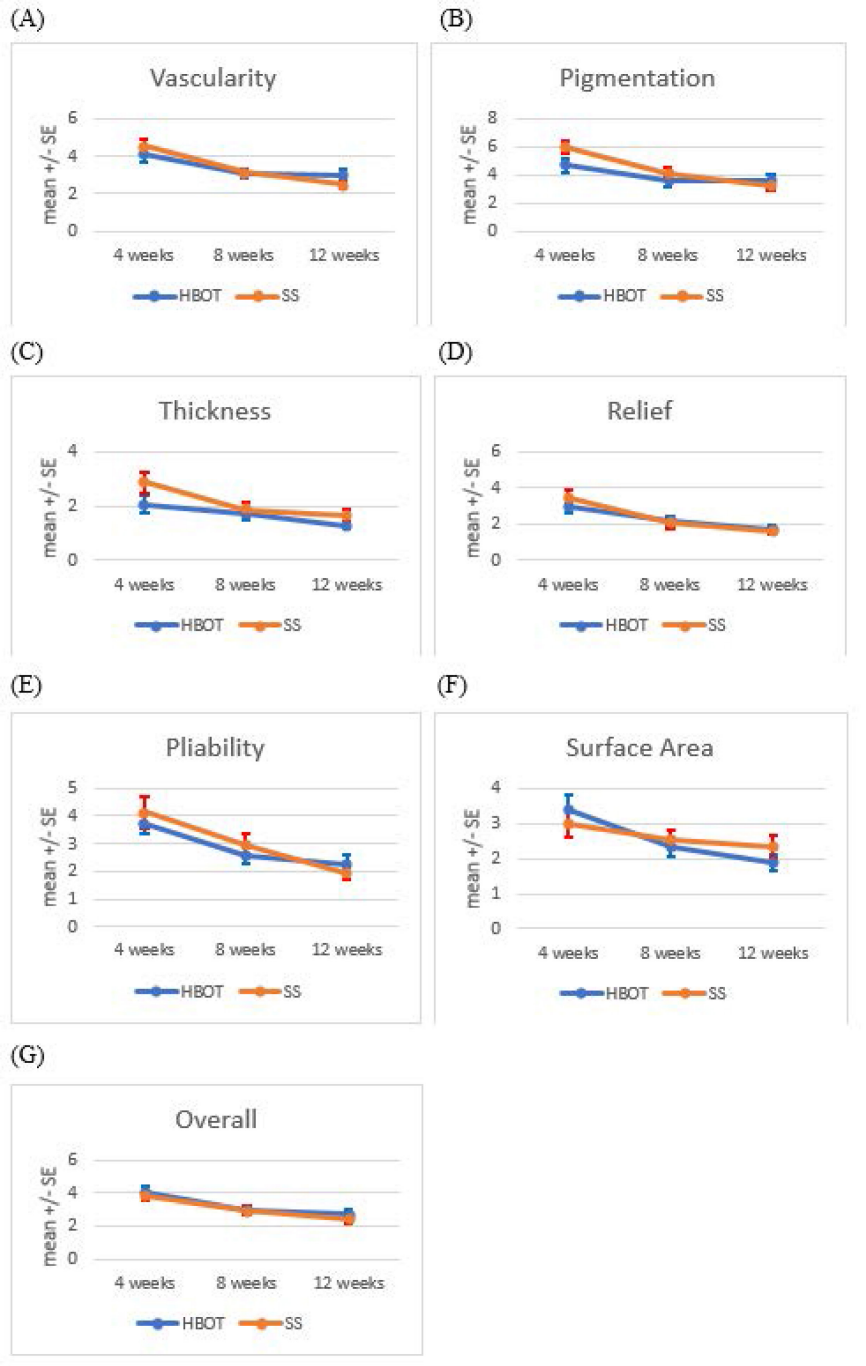
Trends in Patient and Observer Scar Assessment Scale (POSAS) scores over time. POSAS parameters, including vascularity, pigmentation, thickness, relief, pliability, surface area and overall observer opinion, were assessed at 4, 8, and 12 weeks postoperatively in the HBOT and SS groups, illustrating temporal changes in scar characteristics. The data are presented as mean ± standard errors.

Changes in POSAS scores over time for both groups are summarized in Table 2. No significant differences in any parameters were observed between the two groups at any of the three time points. A detailed analysis of each variable, including within-group comparisons and percentage improvements as below are shown in Table 3 and Figure 2 (A-G).

### i. Vascularity

Both groups exhibited significant improvements in vascularity scores at 8 and 12 weeks compared to 4 weeks post-surgery (HBOT group: *p* < 0.01 at 8 weeks, *p* < 0.05 at 12 weeks; SS group: *p* < 0.01 at 8 weeks, *p* < 0.001 at 12 weeks). SS group demonstrating a more percentage improvement (45.8%) compared to the HBOT group (27.0%) in vascularity scores from 4 weeks to 12 weeks post-surgery.

### ii. Pigmentation

The SS group showed a significant reduction in pigmentation scores at 8 and 12 weeks compared to 4 weeks post-surgery (*p* < 0.001 for both time points). The SS group had a greater percentage improvement in pigmentation scores (46.7%) compared to the HBOT group (22.7%) from 4 weeks to 12 weeks post-surgery.

### iii. Thickness

Scar thickness decreased significantly by 12 weeks in the HBOT group compared to 4 weeks post-surgery (*p* < 0.05). In the SS group, significant reductions in thickness were observed at both 8 weeks (*p* < 0.05) and 12 weeks (*p* < 0.01). The percentage improvement in scar thickness was better in the SS group (41.9%) than in the HBOT group (37.9%) from 4 weeks to 12 weeks post-surgery.

### iv. Relief

Relief scores improved significantly within both groups at 8 and 12 weeks compared to 4 weeks post-surgery (HBOT group: *p* < 0.01 at both time points; SS group: *p* < 0.01 at 8 weeks, *p* < 0.001 at 12 weeks). SS therapy resulted in a greater percentage improvement in relief scores (52.9%) compared to HBOT (41.5%) from 4 weeks to 12 weeks post-surgery.

### v. Pliability

Pliability scores improved significantly at 8 and 12 weeks in both groups (HBOT group: *p* < 0.001 at both time points; SS group: *p* < 0.05 at 8 weeks, *p* < 0.001 at 12 weeks). The SS group had a more percentage improvement than HBOT in pliability scores (53.2% vs 38.3%) from 4 weeks to 12 weeks post-surgery.

### vi. Surface area

The HBOT group demonstrated significant reductions in scar surface area at 8 weeks (*p* < 0.01) and 12 weeks (*p* < 0.001) compared to 4 weeks post-surgery. HBOT demonstrated a greater percentage improvement in surface area (44.2%) compared to SS (22.3%) from 4 weeks to 12 weeks post-surgery.

### vii. Overall opinion

Both groups exhibited significant improvements in overall opinion scores at 8 and 12 weeks compared to 4 weeks post-surgery (HBO group: *p* < 0.001 at 8 weeks, *p* < 0.01 at 12 weeks; SS group: *p* < 0.01 at both time points). The SS group showed a slightly higher percentage improvement in overall opinion scores (36.8%) compared to the HBOT group (32.7%) from 4 weeks to 12 weeks post-surgery.

## Discussion

### Mechanisms of action

Understanding the mechanisms underlying HBOT and SS is essential for optimizing their clinical applications. HBOT enhances oxygen delivery to hypoxic tissues, stimulating angiogenesis, facilitating ECM remodeling, and promoting collagen maturation - key processes in wound repair and scar modulation 23-25. By improving tissue oxygenation, HBOT alleviates local ischemia, a contributing factor to delayed healing and excessive scarring 26. Elevated oxygen levels also regulate growth factors and cytokines, enhancing cellular proliferation and tissue regeneration 24.

Silicone therapy primarily functions through occlusion, hydration, cellular signaling modulation, and potential thermoregulation 27, 28. These actions optimize the wound healing environment, reduce scar formation, and improve aesthetic outcomes. By forming a semi-permeable barrier, SS reduces transepidermal water loss (TEWL) and maintains stratum corneum hydration, which is essential for effective epithelialization and scar modulation 29-31. This hydration state attenuates pro-inflammatory cytokine activity 32, 33 and suppresses fibroblast-mediated excessive collagen synthesis, mitigating the risk of hypertrophic scar and keloid formation 34.

### Comparative efficacy of HBOT and SS

Both HBOT and SS demonstrated efficacy in mitigating post-surgical scar formation, albeit with distinct mechanistic advantages. Across POSAS parameters, both modalities yielded comparable improvements in vascularity, thickness, relief, pliability, and overall opinion.

However, differences emerged in specific scar characteristics. The SS group showed significant improvements in pigmentation, a key concern for patients prioritizing aesthetic outcomes. SS's ability to modulate skin hydration and reduce TEWL likely contributes to minimizing pigmentary changes 35. Silicone gel sheeting forms a semi-permeable barrier, maintaining optimal moisture levels, enhancing hydration, and attenuating inflammatory responses 27, 28, which prevent hyperpigmentation and promote cosmetically acceptable outcomes.

In contrast, the HBOT group exhibited superior improvements in scar surface area, highlighting its role in enhancing tissue oxygenation and accelerating wound healing 23-25. HBOT's multifactorial mechanisms include stimulation of angiogenesis, enhanced fibroblast activity, and improved collagen organization, contributing to reduced scar surface area and improved overall scar quality 36. Increased oxygenation during HBOT facilitates collagen synthesis and deposition 37, 38, promoting improved collagen organization and density. These effects result in thinner, more pliable scars 39.

### Implications for clinical practice

Our findings validate the efficacy of HBOT and SS as distinct yet effective interventions for scar prevention. Clinicians should tailor treatment plans based on patient-specific factors, including surgical type, wound characteristics, and individual healing responses. HBOT may be preferable for patients with a history of hypertrophic scars or keloids, particularly when wound healing is impaired by hypoxia 16, 17. In contrast, SS may be ideal for patients seeking noninvasive options or for scars in less mobile areas 27. We posit that engaging patients in discussions about the advantages and limitations of each modality will foster adherence, a key determinant of therapeutic success.

### Timing of treatment initiation

Optimal timing of therapy initiation is critical for maximizing outcomes. While this study initiated treatments at 4 weeks postoperatively, earlier intervention might yield greater benefits, especially for patients at high risk of hypertrophic scarring 40. Evidence suggests that initiating HBOT during the inflammatory phase enhances oxygen delivery to healing tissues 41, potentially mitigating excessive collagen deposition and scarring 40, 42, 43.

Similarly, SS is most effective when applied to fully healed wounds 6, but early initiation within the first few weeks of healing can stabilize the process and minimize pathological scarring 44. Future studies should explore the benefits of initiating both therapies earlier in the postoperative period to determine the optimal timing for scar prevention.

### Considerations for patient adherence

Patient adherence significantly influences the success of scar management strategies. Both HBOT and SS require patient commitment, which various factors, including the perceived efficacy, comfort, and practicality of the treatment, can influence. For instance, HBOT requires multiple sessions in a hyperbaric chamber, posing logistical challenges 45, such as accessibility issues and frequent appointments 46. Clinicians should assess the feasibility of this approach and address potential barriers to compliance.

In contrast, SS offers a more convenient, home-based option with minimal disruption to daily activities. However, adherence may be compromised in areas near joints, where the application is challenging 8, or in highly visible areas (e.g., face) due to aesthetic concerns 27. Educating patients on the importance of consistent use and providing practical guidance can enhance adherence and treatment outcomes.

### Socioeconomic factors

Socioeconomic factors also influence access to scar management therapies. HBOT availability is often limited by geographic location, healthcare infrastructure, and insurance coverage, necessitating travel and time off work 47. SS, being more accessible and cost-effective, is a viable option for a broader patient population.

Addressing these disparities through advocacy for HBOT accessibility and financial support will ensure equitable access to effective scar management.

### Strengths, limitations and future directions

This study contributes to the growing body of evidence on scar management, offering a direct comparison of two widely used interventions. The randomized controlled design strengthens the validity of the findings, reducing bias and ensuring a fair comparison. The use of the POSAS, a validated and comprehensive tool, adds reliability to the scar assessments by considering both objective and subjective perspectives. Furthermore, the inclusion of multiple time points allows for a dynamic evaluation of scar progression over time, providing valuable insights into the temporal effects of each treatment. Despite its strengths, this study has certain limitations that warrant consideration. First, the treatment initiation timeline of 4 weeks post-surgery may have precluded the potential benefits of earlier intervention, especially for HBOT, which could be more effective during the inflammatory phase of wound healing. Future research should explore the outcomes of initiating therapy immediately after wound closure to optimize results. Second, the limited sample size, while sufficient for preliminary findings, restricts the generalizability of the results. Larger studies are needed to validate these findings across diverse populations, particularly among individuals with varying skin types and predispositions to scarring. Additionally, this study focused solely on linear, clean, non-infected wounds, which may not represent the outcomes for more complex or irregular scars, such as hypertrophic or infected wounds. Third, compliance with treatment protocols, particularly in the SS group, could introduce variability in the results. The efficacy of silicone sheets depends heavily on patient adherence, which can be influenced by factors such as discomfort or inconvenience. Evaluating strategies to improve adherence could enhance future outcomes. Fourth, this study did not include a no-treatment control group, limiting the ability to assess the natural course of scar healing. A control group would provide a baseline for evaluating the relative benefits of each intervention. Furthermore, baseline data for scar characteristics at Week 0 were not available, which may be considered a limitation of the study design. This decision was made because the intervention was initiated at Week 4 postoperatively, based on the optimal timing for treatment initiation. Additionally, immediate objective measurements of postoperative wounds (such as vascularity, pigmentation, and pliability) present several challenges. For instance, during the early postoperative period, wounds typically undergo an inflammatory phase characterized by redness, swelling, heat, and pain. These inflammatory responses can alter the wound's appearance, leading to inaccurate measurements. As such, it is generally recommended to perform these measurements during later stages of wound healing to ensure more reliable data. Nevertheless, future studies should consider including baseline measurements at earlier time points, as this data would provide a more comprehensive understanding of the progression of scar characteristics and the relative impact of each treatment modality.

Finally, exploring the combined effects of HBOT and SS remains an intriguing prospect. These treatments address complementary aspects of scar prevention—oxygenation and hydration—which may synergistically reduce hypertrophic scarring and enhance skin pliability. Future studies should investigate this combined approach to uncover potentially superior clinical outcomes.

## Conclusion

HBOT and SS both demonstrate significant efficacy in post-surgical scar prevention, offering distinct advantages through unique mechanisms of action. HBOT excels in improving tissue oxygenation and reducing scar surface area, while SS effectively addresses pigmentation and hydration, making each modality suited to different patient needs. Leveraging these complementary mechanisms allows clinicians to individualize scar management strategies, optimizing both aesthetic and functional outcomes. Future studies should refine treatment protocols, evaluate the synergistic potential of combining HBOT and SS, and investigate the effects of earlier treatment initiation on scar prevention.

## Supplementary Material

Supplementary figure.

## Figures and Tables

**Figure 3:**
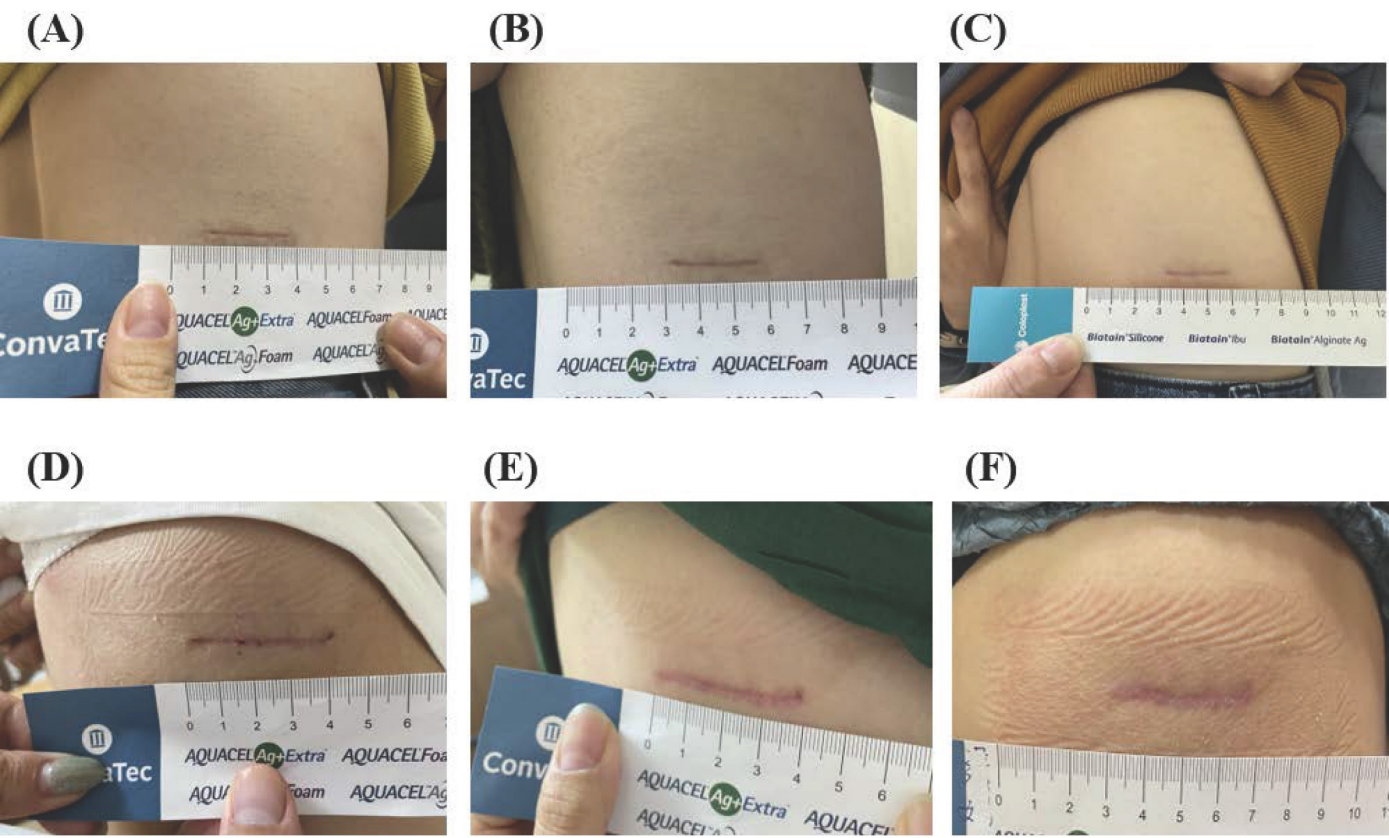
Comparative pre- and post-treatment outcomes in the HBOT and SS groups. (A-C) Representative scar images from the HBOT group at (A) 4 weeks, (B) 8 weeks, and (C) 12 weeks postoperatively. (D-F) Representative scar images from the SS group at (D) 4 weeks, (E) 8 weeks, and (F)12 weeks postoperatively.

**Table 1:** Baseline characteristics of participants

Variable		HBOT group (n=18)	SS Group (n=15)	*p*-value
Age (years)		42.39 ± 13.84	42.87 ± 13.23	0.92
Gender, n (%)				0.77
	Female	10 (55.6%)	10 (66.7%)	
	Male	8 (44.4%)	5 (33.3%)	
Height (m)		1.63 ± 0.07	1.63 ± 0.08	0.993
Weight (kg)		68.22 ± 16.49	63.93 ± 11.68	0.405
Body Mass Index (kg/m^2^)		25.59 ± 5.37	23.94 ± 2.87	0.272
Surgical site, n (%)				0.38
	Face	2 (11.1%)	3 (20.0%)	
	Limbs	9 (50.0%)	4 (26.7%)	
	Trunk	7 (38.9%)	8 (53.3%)	
Wound length (cm)		4.71 ± 3.03	3.33 ± 1.75	0.131

**Table 2:** Variability of POSAS observer scale across groups and time

Parameter	Group	Time (weeks)	Mean ± SD
Vascularity	HBOT	4	4.11 ± 1.64
		8	3.06 ± 1.06**
		12	3.00 ± 1.37*
	SS	4	4.53 ± 1.51
		8	3.13 ± 0.83**
		12	2.47 ± 0.92***
Pigmentation	HBOT	4	4.67 ± 2.09
		8	3.61 ± 1.85
		12	3.61 ± 1.82
	SS	4	6.00 ± 1.65
		8	4.13 ± 1.51***
		12	3.20 ± 1.15***
Thickness	HBOT	4	2.06 ± 1.31
		8	1.72 ± 0.90
		12	1.28 ± 0.58*
	SS	4	2.87 ± 1.51
		8	1.87 ± 0.99*
		12	1.67 ± 0.90**
Relief	HBOT	4	2.94 ± 1.31
		8	2.17 ± 0.99**
		12	1.72 ± 1.13**
	SS	4	3.40 ± 1.72
		8	2.07 ± 1.10**
		12	1.60 ± 0.74***
Pliability	HBOT	4	3.72 ± 1.64
		8	2.56 ± 1.20***
		12	2.28 ± 1.27***
	SS	4	4.13 ± 2.13
		8	2.93 ± 1.58*
		12	1.93 ± 0.80***
Surface Area	HBOT	4	3.39 ± 1.69
		8	2.33 ± 1.19**
		12	1.89 ± 0.96***
	SS	4	3.00 ± 1.51
		8	2.53 ± 1.13
		12	2.33 ± 1.23
Overall	HBOT	4	4.06 ± 1.31
		8	2.94 ± 0.80***
		12	2.72 ± 1.49**
	SS	4	3.80 ± 1.08
		8	2.93 ± 1.10**
		12	2.40 ± 0.91**

^*^p < 0.05, ^**^p < 0.01,^ ***^p < 0.001 compared to 4 months (within-group comparisons) ^#^*p* < 0.05, ^##^*p* < 0.01, ^###^*p* < 0.001, compared to the HBOT group (between-group comparisons0)

**Table 3:** Percentage improvements of parameters (from 4 weeks to 12 weeks)

Parameter	Group	Percentage Improvement (%)
Vascularity	HBOT	-27.00%
	SS	-45.80%
Pigmentation	HBOT	-22.70%
	SS	-46.70%
Thickness	HBOT	-37.90%
	SS	-41.90%
Relief	HBOT	-41.50%
	SS	-52.90%
Pliability	HBOT	-38.30%
	SS	-53.20%
Surface Area	HBOT	-44.20%
	SS	-22.30%
Overall	HBOT	-32.70%
	SS	-36.80%

## Abbreviations

SS: silicone sheets
HBOT: hyperbaric oxygen therapy
ECM: extracellular matrix
POSAS: Patient and Observer Scar Assessment Scale
