# Supplementary material for: Comparative Efficacy of Silicone Sheets and Hyperbaric Oxygen Therapy in Post-Surgical Scar Prevention: A Prospective Observational Study
Source: Int J Med Sci. 2025 Feb 11;22(5):1167–75. doi: 10.7150/ijms.108397 (PMC11866543; doi:10.7150/ijms.108397)
Supplement: Supplementary file 1 — Supplementary figure. [file ijmsv22p1167s1.pdf]

Supplementary Data

Supplementaty Figure 1. Patient and Observer Scar Assessment Scale (POSAS) 2.0 Observer Scale

POSAS Observer scale

The Patient and Observer Scar Assessment Scale v 2.0 / EN

**Date of examination:**	**Name of patient:**
**Observer:**	
**Location:**	**Date of birth:**
**Research / study:**	**Identification number:**

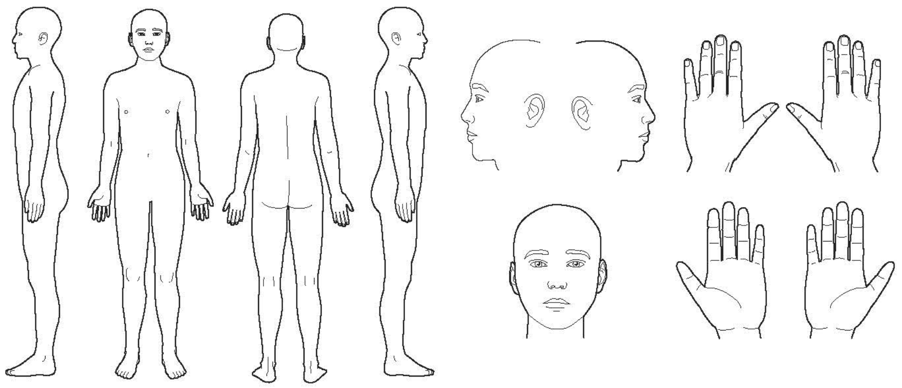

1 = normal skin **worst scar imaginable = 10**

PARAMETER	1	2	3	4	5	6	7	8	9	10	CATEGORY
VASCULARITY											PALE | PINK | RED | PURPLE | MIX
PIGMENTATION											HYPO | HYPER | MIX
THICKNESS											THICKER | THINNER
RELIEF											MORE | LESS | MIX
PLIABILITY											SUPPLE | STIFF | MIX
SURFACE AREA											EXPANSION | CONTRACTION | MIX
OVERALL OPINION											

Explanation

The observer scale of the POSAS consists of six items (vascularity, pigmentation, thickness, relief, pliability and surface area).
All items are scored on a scale ranging from 1 ('like normal skin') to 10 ('worst scar imaginable').
The sum of the six items results in a total score of the POSAS observer scale. Categories boxes are added for each item. Furthermore, an overall opinion is scored on a scale ranging from 1 to 10.
All parameters should preferably be compared to normal skin on a comparable anatomic location.

Explanatory notes on the items:

- **VASCULARITY** Presence of vessels in scar tissue assessed by the amount of redness, tested by the amount of blood return after blanching with a piece of Plexiglas
- **PIGMENTATION** Brownish coloration of the scar by pigment (melanin); apply Plexiglas to the skin with moderate pressure to eliminate the effect of vascularity
- **THICKNESS** Average distance between the subcutical-dermal border and the epidermal surface of the scar
- **RELIEF** The extent to which surface irregularities are present (preferably compared with adjacent normal skin)
- **PLIABILITY** Suppleness of the scar tested by wrinkling the scar between the thumb and index finger
- **SURFACE AREA** Surface area of the scar in relation to the original wound area

COPYRIGHT © P.P.M. VAN ZUIJLEN, BEVERWIJK-NL
